# Fine-scale genetic correlates to condition and migration in a wild cervid

**DOI:** 10.1111/eva.12189

**Published:** 2014-08-28

**Authors:** Joseph M Northrup, Aaron B A Shafer, Charles R Anderson, David W Coltman, George Wittemyer

**Affiliations:** 1 Department of Fish, Wildlife, and Conservation Biology, Colorado State UniversityFort Collins, CO, USA; 2 Department of Evolutionary Biology, Evolutionary Biology Centre, Uppsala UniversityUppsala, Sweden; 3 Mammals Research Section, Colorado Parks and WildlifeGrand Junction, CO, USA; 4 Department of Biological Sciences, University of AlbertaEdmonton, AB, Canada

**Keywords:** genetic differentiation, heterozygosity fitness correlation, migration, mule deer, multilocus heterozygosity, *Odocoileus hemionus*, single-locus heterozygosity, wildlife

## Abstract

The relationship between genetic variation and phenotypic traits is fundamental to the study and management of natural populations. Such relationships often are investigated by assessing correlations between phenotypic traits and heterozygosity or genetic differentiation. Using an extensive data set compiled from free-ranging mule deer (*Odocoileus hemionus*), we combined genetic and ecological data to (i) examine correlations between genetic differentiation and migration timing, (ii) screen for mitochondrial haplotypes associated with migration timing, and (iii) test whether nuclear heterozygosity was associated with condition. Migration was related to genetic differentiation (more closely related individuals migrated closer in time) and mitochondrial haplogroup. Body fat was related to heterozygosity at two nuclear loci (with antagonistic patterns), one of which is situated near a known fat metabolism gene in mammals. Despite being focused on a widespread panmictic species, these findings revealed a link between genetic variation and important phenotypes at a fine scale. We hypothesize that these correlations are either the result of mixing refugial lineages or differential mitochondrial haplotypes influencing energetics. The maintenance of phenotypic diversity will be critical to enable the potential tracking of changing climatic conditions, and these correlates highlight the need to consider evolutionary mechanisms in management, even in widely distributed panmictic species.

## Introduction

Understanding variation in phenotypic traits related to fitness in wild populations is fundamental to the study of evolution and ecology. Such traits can be related to genetic variation at relatively fine spatial scales, and knowledge of these relationships can provide insight into important eco-evolutionary processes such as inbreeding depression, local adaptation, population structure, and speciation (Kupper et al. 2010; Olano-Marin et al. 2011; Shafer and Wolf 2013; Shafer et al. 2014). Moreover, these relationships can have implications for developing and implementing conservation and management plans that strive to account for evolutionary processes (e.g., maintenance of gene flow through protection of corridors or minimizing possible effects of inbreeding).

Relationships between fine-scale genetic variation and phenotypic traits have been identified using a variety of methods. Chief among these in wild populations are heterozygosity–fitness correlations (HFCs; see Chapman et al. 2009) and correlations among genetic differentiation and phenotypic or ecological divergence (Shafer and Wolf 2013). Heterozygosity–fitness correlations are typically calculated between heterozygosity at neutral loci and phenotypic traits presumed to be proxies for fitness (Szulkin et al. 2010). Correlations can occur with a multilocus heterozygosity (MLH) metric, indicating a general genome-wide effect of inbreeding, or heterozygosity at a single locus (single-locus heterozygosity; SLH), indicating local (either direct or indirect) effects due to linkage to a gene that affects fitness (Hansson et al. 2004). For the latter, individual neutral markers are hypothesized to show associative overdominance as a result of the consequences of deleterious alleles or a fitness advantage at those linked loci (Frydenberg 1963; Houle 1989; David et al. 1995; David 1997; Pamilo and Palsson 1998). Screening for HFCs can be described as a tantalizing pursuit; significant relationships are rarely found and care must be used with interpretation as overall effect sizes often are variable and small (Chapman et al. 2009; Kardos et al. 2014), and numerous concerns (but also caveats) related to the HFC exist (Szulkin et al. 2010). Given the potential for false positives with SLH correlations, confidence in these relationships can be bolstered by appropriate statistical analyses and by examining the location of loci on the annotated genome of a related species that might provide *post hoc* links to causative agents (Von Hardenberg et al. 2007; Kupper et al. 2010; Kardos et al. 2014).

In slight contrast, correlations between genetic differentiation and phenotypic (or ecological) divergence have been identified across taxa and appear to be relatively robust (Shafer and Wolf 2013; Sexton et al. 2014). While this pattern is generally regarded as evidence for local adaptation (Nosil 2012), ancestral (allopatric) divergence and secondary contact can confound interpretations of this correlation (Bierne et al. 2013) and, similar to HFCs, must be factored into interpretations and models. But beyond these caveats, correlations between phenotypic traits and both genetic diversity and differentiation can provide important indications of inbreeding and local adaptation that should be considered by managers (Shafer et al. 2014).

### Mule deer ecology and evolution

Cervids (family *Cervidae*) are an ecologically important group of ungulate that have been the focus of numerous investigations into the relationship between genetic variation and phenotypic traits. Da Silva et al. (2009) showed that juvenile roe deer (*Capreolus capreolus* L.) survival was correlated with MLH; likewise, red deer (*Cervus elaphus* L.) birth weight, neonatal survival, and lifetime breeding success increased significantly with heterozygosity (Coulson et al. 1998; Slate et al. 2000), and individuals with the smallest antlers tended to have lower heterozygosity (Perez-Gonzalez et al. 2010). Furthermore, studies have shown correlations between genetic differentiation and social groups in white-tailed deer (*Odocoileus virginianus* Zimm.; Miller et al. 2010), and niche overlap in mule deer (*Odocoileus hemionus* Raf.; Pease et al. 2009).

Among cervids, mule deer present an interesting species for which to examine correlations between phenotypic traits and genetic variation. Latch et al. (2009, 2014) showed that across their range, there are multiple phylogeographic lineages that presumably represent different refugia, although the species shows minimal population-level genetic structure at large geographic scales (Cullingham et al. 2011b; Powell et al. 2013). Female mule deer also display fine-scale genetic structuring, likely due to the existence of related social groups (Cullingham et al. 2011b; Colson et al. 2013). In addition, hybridization with white-tailed deer can occur, resulting in fairly widespread genetic introgression (Carr et al. 1986; Cathey et al. 1998). Mule deer also exhibit substantial variation in important phenotypic traits such as body size and migratory behavior, both across their range (Anderson 1981; Wallmo 1981), and within populations (Monteith et al. 2011a; Lendrum et al. 2013). Lastly, mule deer are the subject of extensive management programs throughout North America, due to their importance as a game species [e.g., it was estimated that more than 30 000 mule deer were harvested in the state of Colorado in 2013 (Colorado Parks and Wildlife 2014)].

Both the aforementioned phenotypic traits are of paramount importance for survival and reproduction in this species. Condition is a fitness proxy as individuals rely heavily on fat and protein stores for survival on winter range when forage quality is low (Wallmo et al. 1977; Torbit et al. 1985). Body fat also influences annual survival of adult females (Bender et al. 2007), pregnancy and twinning rates (Johnstone-Yellin et al. 2009; Tollefson et al. 2010), and the probability of a female rearing a fawn through the summer (Johnstone-Yellin et al. 2009). Deer across much of their range migrate from high altitude, productive summer range to low altitude winter range and back again in the spring. Migrations typically match changes in resource availability (Fryxell and Sinclair 1988), with mule deer attempting to optimize migratory timing relative to both plant productivity and weather (snow depth and temperature) on their summer range (Monteith et al. 2011a; Lendrum et al. 2013). The timing of migratory onset is clustered around a few weeks each year, but individuals show different strategies in terms of early or late onset dates (Monteith et al. 2011a; Lendrum et al. 2013). Thus, migration timing is of clear interest in understanding the ecology of this species and, importantly, recent work has identified a clear genetic component to differences in this trait in other taxa (Ruegg et al. 2014; Toews et al. 2014).

Both individual condition and migration are of interest to wildlife managers as recent anthropogenic development may threaten migratory routes for mule deer (Sawyer et al. 2005, 2009), and climate change could cause trophic mismatches (Post and Forchhammer 2008), with phenotypic plasticity in migration being suggested as a potential buffer for mule deer against this process (Monteith et al. 2011b). The importance of winter condition to deer survival has led to active research into means of improving winter condition through habitat manipulation and supplemental feeding (Bishop et al. 2009; Bergman et al. 2014). The existence of genetic correlations to these traits could provide insight into the effectiveness of management programs and aid managers in making decisions in light of evolutionary processes.

Here, we examined the relationship between genetic variability and phenotypic traits in a wild mule deer population of the Piceance Basin, Colorado. Using an extensive data set consisting of more than 100 individual animals, we combined phenotypic, behavioral (global positioning system [GPS]), and genetic data to (i) examine whether genetic differentiation was correlated with migration timing, (ii) screen for specific mitochondrial haplotypes associated with migration timing, and (iii) test whether heterozygosity (multilocus and single locus) was associated with body mass and fat. We discussed the results in light of the phylogeographic history of mule deer and the metabolic role of the mitochondrion and highlight the importance of considering evolutionary processes in the management of this species.

## Materials and methods

### Sample collection and DNA extraction

**Figure 1:**
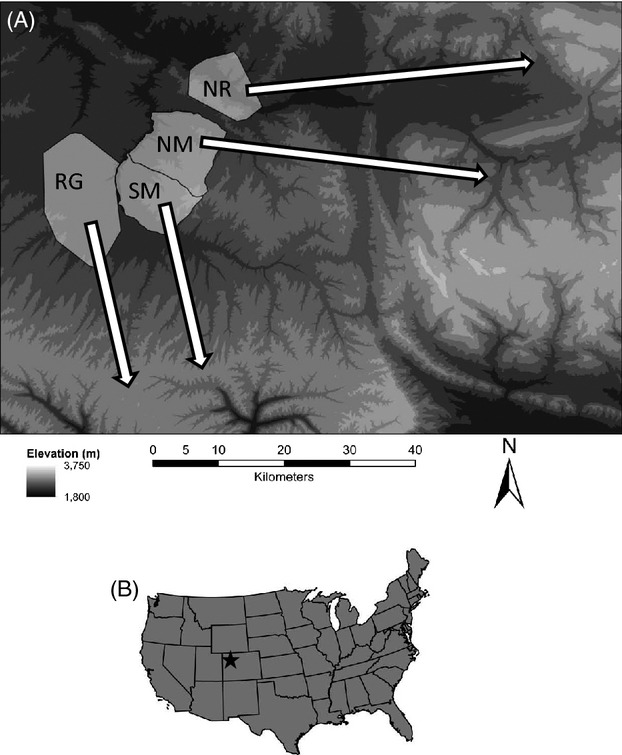
(A) Winter range areas Ryan Gulch [RG], South Magnolia [SM], North Magnolia [NM] and North Ridge [NR]) and simplifications of migratory routes, with arrows indicating general location of summer ranges for mule deer in the Piceance Basin and (B) location of study within the United States. Adapted with permission from Lendrum et al. (2013).

We captured adult (>1 year old) female mule deer using helicopter net gunning in four winter range study areas in the Piceance Basin of Northwestern Colorado (Fig. 1). Deer were captured in either December 2010 or March 2011. These dates were chosen because during December, deer have recently migrated from summer range and typically are in their best physical condition, while March represents the end of winter when deer typically are in their worst condition. Deer were transferred to processing sites where we weighed them using a portable scale, estimated body condition by palpating the rump (Cook et al. 2001, 2007, 2010) and measured the thickness of their subcutaneous rump fat and longissimus dorsi muscle using a portable ultrasound (Stephenson et al. 1998, 2002; Cook et al. 2001). The above measurements were used to calculate the percent ingesta-free body fat (hereafter fat) of each deer following Cook et al. (2010). Deer were fit with store-on-board GPS radio collars (Advanced Telemetry Systems, Isanti MN, USA) set to attempt a relocation on one of three schedules (once every 5 h, once every 60 min, or once every 30 min – meaning the relocation schedules varied by individual). Blood samples were taken for genetic analysis, and DNA was extracted using the DNeasy™ Blood and Tissue Kit (Qiagen, Inc., Valencia, CA, USA) following the manufacturer's protocol.

### Microsatellite genotyping and DNA sequencing

We amplified 17 microsatellite loci using a previously optimized multiplex reaction from Cullingham et al. (2011a) and single PCRs. The mitochondrial control region was sequenced using both the primers from Latch et al. (2009) and LGL215 and ISM015 from Purdue et al. (2006). PCR conditions and basic population genetic analyses are available in Appendices S1 and S2.

For the microsatellite data, we first used structure 2.3.3 (Pritchard et al. 2000) to assess genetic structure (1 000 000 iterations with 25% removed as a burn-in repeated five times for each number of possible populations [*k*] ranging from 1 to 5). We assumed an admixed model with correlated allele frequencies (Falush et al. 2003) and used the LOCPRIOR parameter to allow location information to assist in the clustering. Next, we calculated overall MLH as the average of heterozygosity at each locus and SLH as binary variables indicating heterozygosity (1) or homozygosity (0) at each locus. Pairwise relatedness between all individuals was estimated with the Queller and Goodnight (QG) relationship coefficient using the software spagedi v.1.3 (Hardy and Vekemans 2002). We also constructed a coancestry matrix using the software mol_coan v.3 (Fernandez and Toro 2006). Here, a simulated annealing approach was used to create virtual common ancestors of the genotyped individuals, producing pedigree-like relationship coefficients. Model parameters consisted of 200 steps with 5000 solutions tested per step, an initial temperature of 0.01 and increase of 0.75. We simulated two previous generations, each consisting of 1000 males and 1000 females.

For mitochondrial DNA (mtDNA; conducted on a subset of individuals), we constructed a minimum-spanning tree among haplotypes using arlequin v. 3.5.1.3 (Excoffier and Lischer 2010) and edited it with hapstar v0.7 (Teacher and Griffiths 2011). Neighbor-joining analysis using pairwise deletion and both *P* and *K2* distances was conducted using the software package mega v.5 (Tamura et al. 2011). Bayesian analysis was conducted in mrbayes v.3.1.2 (Huelsenbeck and Ronquist 2001) with a model of nucleotide substitution determined from modeltest v.3.07 (Posada and Crandall 1998). For the Bayesian phylogenetic analysis, we used default priors with two independent runs of four chains (three heated) run for 10 000 000 generations, with the first 25% discarded as a burn-in. Confidence in topologies was evaluated based on 1000 bootstrap replicates (for the neighbor joining) or posterior distributions. All three methods were compared to identify common mitochondrial haplogroups.

### Genetic correlates to phenotypic traits

Both migration and body condition are phenotypic traits that are important to the fitness of mule deer. However, only condition can be thought of as a proxy for fitness. Thus, we used two separate analytical frameworks to examine genetic correlations with these traits. For migration, we examined the relationship between mitochondrial haplotypes and genetic differentiation to determine whether there was a genetic component to the timing of migration (an isolation-by-ecology analysis, *sensu* Shafer and Wolf 2013). For body condition, a fitness proxy, we followed the general HFC framework discussed in Chapman et al. (2009).

#### Genetic–migration correlates

After GPS radio collars were recovered and data were downloaded, we calculated the initiation and termination dates of spring and fall migration (i.e., the dates at which deer started or finished their migration) in arcmap 10.1 (Environmental Systems Research Institute, Redlands, CA, USA). Migration was demarcated as the time period during which deer travelled between their winter and summer home ranges. Home ranges were determined by outlining a minimum convex polygon around all locations that occurred prior to directed movement, without return, away from the summer or winter range areas.

We first examined the relationship between mtDNA haplogroup (derived from haplotype and phylogenetic analyses) and the dates of spring and fall migrations. For this analysis, we corrected the Julian date of migration to the earliest date among all individuals. The resulting data represented a count of the number of days since the earliest arriving or leaving migrant had terminated or initiated their migration. These data were analyzed using negative binomial regression (see Appendix S3 for model formulation). We included covariates for the mtDNA haplogroup to which each deer was assigned (categorical) as well as a covariate for the age of the animal and binary covariates indicating winter range study area (i.e., three separate covariates indicating whether the deer was from a winter range study area [1] or not [0]). Before models were run, correlations among covariates were examined to assess collinearity (no predictors were correlated at |*r*| > 0.7) and age was standardized 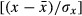, a common procedure in regression to aid in interpretability of coefficient estimates (Gelman and Hill 2007). We fitted all models under a Bayesian framework in JAGS (Plummer 2012) and r3.0.1 (R Core Team 2013), using the ‘rjags’ package (Plummer 2013). See Appendix S3 for specifics of model runs and assessment of convergence. To assess the fit of the models, we calculated residuals (observed – predicted values) and plotted them against the fitted values to examine any potential patterns in residuals.

Secondly, we examined correlations between genetic relatedness metrics and similarity in migration using Mantel tests. For this analysis, we calculated absolute pairwise distances (calculated in days) between each individual's migration termination or initiation dates leaving us with four matrices representing differences in migration timing for spring and fall. The relationships between relatedness indices (QG and coancestry) and migratory behavior (dates) were evaluated in r3.0.1 (R Core Team 2013) using Mantel tests (Mantel 1967) under 10 000 permutations as implemented by the Ecodist library (Goslee and Urban 2007). Here, a comparison is made between relatedness and the difference in migration timing, and thus, a negative relationship is expected if there is a genetic signature – that is, more closely related individuals have more similar migration timing. To account for similarities among individuals inhabiting similar areas or grouping together, we ran two partial Mantel tests controlling for the distance between the centroids of individuals' winter range and summer range (Fig. 1). Significance was assessed by examining 95% confidence intervals.

#### Genetic–condition correlates

We next examined whether there was a relationship between either MLH or SLH and condition metrics (mass and fat) using the HFC framework. We fit hierarchical (i.e., random effects) models in a Bayesian framework. The presence or the absence of a relationship was determined by examining the posterior probability distributions of each coefficient to determine the probability that either MLH or heterozygosity at any single locus was related to condition. In all models, we included covariates for either MLH or SLH, the age of the animal, a binary variable for if the data came from a March capture (both mass and fat are expected to be lower in March), and binary variables indicating which of the four winter range areas the deer was captured in (as in the migration analysis, above). We tested between models with solely a linear effect or a quadratic effect of age using the deviance information criteria (DIC; Spiegelhalter et al. 2002; but with the effective number of parameters calculated as in Plummer 2012). Identity disequilibrium among loci (i.e., covariance of heterozygosity among loci) was used to infer the validity of MLH correlation: Accordingly, we calculated *g*^2^, where a value of zero means no variance in inbreeding (Szulkin et al. 2010).

We examined the relationship between heterozygosity and mass or fat using linear regression and beta regression, respectively. Mass was natural log-transformed to ensure proper support (i.e., untransformed mass is strictly positive, while linear regression allows for negative values; log transformation addresses this issue), while beta regression was used because it is proper for dependent variables ranging between 0 and 1 as percent body fat does. Because there were multiple condition measures for certain deer (i.e., those captured in both March and December), for both analyses, we allowed the intercept to vary by individual, estimating a population-level intercept (i.e., we fit a random intercept by individual), with all other coefficient values fixed. See Appendix S3 for details of model parameters and convergence assessment. To assess the fit of the models, we calculated residuals (observed – predicted values) and plotted them against the fitted values to examine any potential patterns in residuals.

## Results

### Genotype and mitochondrial sequence data

**Figure 2:**
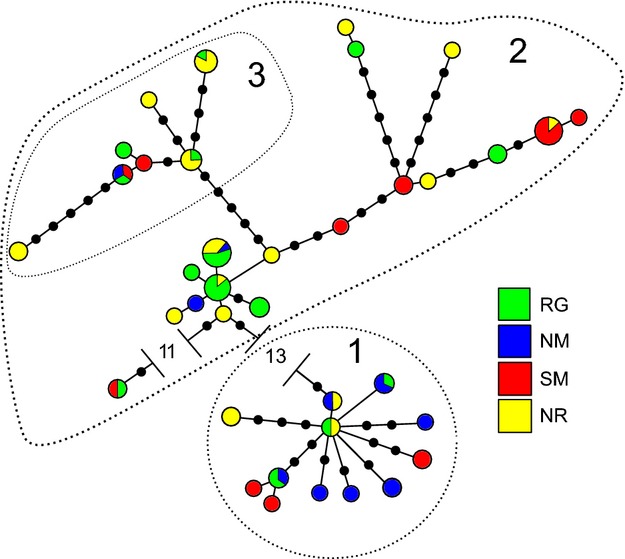
Mitochondrial control region haplotype network and winter range area assignments. Circle size is proportional to the haplotype frequency with small black circles representing undetected, intermediate haplotypes. Haplotypes are colored according to winter range area. The dashed circle outlines and corresponding numbers are in reference to the phylogenetic clades (Appendix S4).

A total of 134 adult female deer were captured with 30 captured in the NM area, 30 in the NR area, 44 in the RG area, and 30 in the SM area (102 in December, and 79 in March, with 47 caught during both capture periods; see Appendix S5 for details). Deer ranged in age from yearlings to more than 11 years old, with a median age of 5.5 years old (See Appendix S5). All 134 deer were genotyped at 17 loci producing a data set that was 99% complete (data available from the Dryad Digital Repository: http://datadryad.org/resource/doi:10.5061/dryad.3vc1b). All markers were in Hardy–Weinberg Equilibrium, and there was no evidence of linkage (diversity statistics by loci are presented in Appendix S2). The structure-based analysis of the microsatellites suggested a single, homogenous population was most likely (i.e., had the lowest likelihood score). Based on winter range, *F*_IS_ values were as follows: NR = −0.05 (*P* = 0.02), NM = −0.02 (*P* = 0.16), RG = −0.03 (*P* = 0.07), and SM = 0.01 (*P* = 0.31). The mol_coan analysis produced a matrix of pedigree-like coefficients for all individuals; we note the one suspected mother–daughter pairing had a coefficient of 0.50, suggesting the results were indeed reflective of pedigree data. We sequenced the mitochondrial control region in a subset of animals (*n* = 81). For comparison with data from Latch et al. (2009), we parsed the data set down to 545 base pairs (GenBank submission KM061069–KM061151). Examining the mtDNA, 37 unique haplotypes were observed (Fig. 2). The neighbor-joining and Bayesian phylogeny (based on a GTR + I + G substitution model) produced essentially the same topology (Appendix S4): a major split between two clades was highly supported, while a third, more tenuous clade was evident in the neighbor analysis with some support in the Bayesian analysis (posterior probability = 0.60). The three groupings are identified in the haplotype network (Fig. 2).

### Genetic correlates to phenotypic traits

#### Condition and migration data

We obtained mass and fat measures on 134 adult female mule deer. Migration data were not obtained for all deer due to mortalities, collar failure, or because some deer were not collared during capture. Thus, our total sample for microsatellites analyses examining relationships with migration consisted of 104 and 95 deer for spring and fall migration, respectively. Our total sample for mtDNA analyses consisted of 65 and 59 deer for spring and fall migration, respectively. In addition, two deer did not leave summer range, while collars were still attached and thus were excluded from the fall migration analyses. During spring, deer initiated migration between April 11 and June 1 and terminated migration between April 19 and June 21. During the fall, deer initiated migration between October 4 and November 8 and terminated migration between October 6 and November 14.

#### Genetic–migration correlates

For all regression models, hereafter, we made inference based on the proportion of the posterior distributions that fell to one side of 0. Winter range area was related to fall migration termination and initiation dates, while age was not related to migration timing in any of the analyses (Table 1; Appendix S5). The mtDNA haplogroups were related to both fall termination and initiation, although both the effect itself and the probability of an effect were lower for fall initiation (Table 1; Appendix S5). For haplogroups identified by the Bayesian phylogenetic analysis, our models predicted that deer in haplogroup 2 terminated migration 6 days earlier on average than those in haplogroup 1 (see Fig. 2 for haplogroups), while for the neighbor-joining analysis, models predicted that deer in haplogroups 2 and 3 terminated migration on average 7 and 9 days earlier than those in haplogroup 1. Plots of residuals against fitted values showed no trend, although the six largest negative residuals were all from the NR winter range area, indicating the potential for a missing covariate (Appendix S5). The microsatellites analyses showed that related individuals generally migrated at similar times regardless of the distance between them on summer or winter range (Table 2).

**Table 1:** Covariates, median coefficient (coeff.) values, and the probability (prob.) of either a negative or positive effect of the covariate from negative binomial regression model on mule deer fall migration termination dates from deer in the Piceance basin, Colorado

Covariate	Median coeff. value	Prob. coeff. is negative	Prob. coeff. positive
Neighbor-joining clades			
Intercept	3.08	0.00	1.00
Age	−0.09	0.88	0.12
Winter range			
NR*	0.16	0.22	0.78
RG†	−0.38	0.96	0.04
SM‡	−0.56	0.99	0.01
mtDNA			
Haplogroup 2§	−0.46	0.99	0.01
Haplogroup 3§	−0.33	0.94	0.06
Bayesian clades			
Intercept	2.932	0.000	1.000
Age	−0.095	0.90	0.10
Winter range			
NR*	0.1768	0.22	0.78
RG†	−0.270	0.90	0.10
SM‡	−0.440	0.97	0.03
mtDNA			
Haplogroup 2§	−0.350	0.97	0.03

* Deer captured in the NR winter range, with NM as the reference category.

† Deer captured in the RG winter range, with NM as the reference category.

‡ Deer captured in the SM winter range, with NM as the reference category.

§ mtDNA haplogroup 1 is the reference category.

**Table 2:** Mantel test models, Mantel's *r* and lower and upper confidence limits (CL), calculated through randomization, for models examining correlation between relatedness metrics (Queller-Goodnight [QG] and coancestry) and migration dates, for mule deer in the Piceance basin, Colorado. End spring and end fall indicate the termination of spring and fall migration, respectively. Start spring and start fall indicate the initiation of spring and fall migration, respectively. Winter distance and summer distance indicate the distance between winter and summer range centroids. All values are presented as Mantel *r* (lower CL, upper CL). Vertical lines (|) indicate partial Mantel tests with the covariate that is controlled for following the vertical line

Migratory metric	QG	Coancestry
End spring	−0.04 (−0.06, −0.01)	−0.06 (−0.09, −0.01)
End spring | winter distance	−0.02 (−0.04, −0.001)	−0.07 (−0.10, −0.03)
End spring | summer distance	−0.03 (−0.05, −0.01)	−0.05 (−0.08, −0.02)
End fall	−0.04 (−0.06, −0.01)	−0.02 (−0.05, 0.02)
End fall | winter distance	−0.04 (−0.06, −0.01)	−0.02 (−0.05, 0.02)
End fall | summer distance	−0.04 (−0.06, −0.01)	−0.02 (−0.05, 0.01)
Start spring	0.002 (−0.02, 0.02)	−0.03 (−0.07, 0.01)
Start spring | winter distance	0.01 (−0.02, 0.02)	−0.03 (−0.06, 0.01)
Start spring | summer distance	0.01 (−0.01, 0.04)	−0.02 (−0.06, 0.01)
Start fall	−0.05 (−0.07, −0.03)	−0.05 (−0.08, −0.03)
Start fall | winter distance	−0.05 (−0.06, −0.01)	−0.05 (−0.08, −0.03)
Start fall | summer distance	−0.05 (−0.07, −0.03)	−0.05 (−0.08, −0.03)

### Genetic–condition correlates

**Figure 3:**
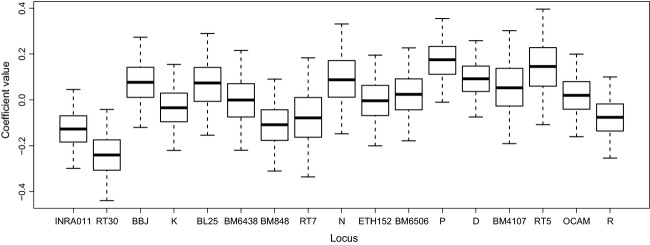
Box plots of coefficients for effect of microsatellite loci on mule deer body fat percent. Coefficients were obtained through beta regression model in a Bayesian hierarchical framework. Box plots represent median (black line) interquartile range (box bounds) and upper and lower 95% bounds (whiskers) of coefficient values.

There was weak evidence for identity disequilibrium (*g*^2^ = 0.01, *P* = 0.07); however, MLH was a poor predictor of both body mass and fat in all models (Appendix S5 Table 2), while heterozygosity at individual loci was strongly related to condition measures (Table 3; Appendix S5 Table 2). Because heterozygosity at individual loci was the only significant correlates to the phenotypic traits, we continued with this model only. When examining the relationship between SLH and body mass, models with a quadratic term for age fit the data slightly better than those with a linear term, with evidence for greater body mass for middle aged deer compared with young or old deer (Appendix S5 Table 2). When examining fat, models with a linear effect of age fit the data slightly better, and age was a poor predictor of fat (Table 3; Appendix S5 Table 2). Winter range area was weakly related to both body mass and fat (<95% of posterior on one side of 0; Table 3; Appendix S5 Table 2). Heterozygosity at two loci (RT30 and P) was strongly related to fat (>95% of posterior on one side of 0; Table 3; Fig. 3). Plots of residuals against fitted values showed a positive trend, with all of the largest fitted values showing positive residuals (Appendix S5). To guard against false positives, we refit the models with a strong mean 0 multivariate normal prior on the coefficients, which shrinks coefficient estimates toward 0 (the standard deviation on the prior was taken as the standard deviation of the median coefficient values; approximately 0.14; Gelman et al. 2012).

**Table 3:** Covariates, median coefficient (coeff.) values, and the probability (prob.) of either a negative or positive effect of the covariate from multilevel beta regression on the percent body fat of mule deer in the Piceance basin, Colorado

Covariate	Median coeff. value	Prob. coeff. is negative	Prob. coeff. positive
Intercept	−2.15	1	0
Age	−0.05	0.89	0.11
March capture	−0.52	1	0
Winter range			
NR*	−0.10	0.80	0.20
RG†	−0.11	0.82	0.18
SM‡	−0.06	0.69	0.31
Microsatellite loci			
INRA011	−0.13	0.93	0.07
RT30	−0.24	0.99	0.01
BBJ	0.08	0.22	0.78
*K*	−0.03	0.65	0.35
BL25	0.07	0.27	0.73
BM6438	−0.001	0.50	0.50
BM848	−0.11	0.87	0.13
RT7	−0.08	0.72	0.28
*N*	0.09	0.22	0.78
ETH152	−0.004	0.52	0.48
BM6506	0.02	0.40	0.60
*P*	0.18	0.04	0.96
*D*	0.092	0.13	0.87
BM4107	0.05	0.32	0.68
RT5	0.15	0.13	0.87
OCAM	0.02	0.41	0.59
*R*	−0.08	0.81	0.19

* Deer captured in the NR winter range, with NM as the reference category.

† Deer captured in the RG winter range, with NM as the reference category.

‡ Deer captured in the SM winter range, with NM as the reference category.

## Discussion

We documented relationships between phenotypic traits recognized as being critical to fitness and genetic variation at a very fine spatial scale in female mule deer. These results provide insight into the genetic structuring of the population and the possible genetic drivers shaping the diversity of phenotypes and migration strategies seen in this important game species. These findings have potential implications for conservation and management, particularly in light of contemporary climatic changes and white-tailed deer expansion (Latham et al. 2011), as both migration timing and body condition are influential traits for mule deer survival and reproduction that vary among individuals in a population (Monteith et al. 2011b, 2013). Examining these traits conjointly provided a more complete picture of the genetic contributions to important phenotypic traits in this population and cervids in general.

### Genetic-migration correlations

Fall and spring migration dates were more similar among related females. An individual's mtDNA haplogroup also was a stronger predictor of fall than spring migration – even when controlling for winter or summer range. The mtDNA haplotype effect is particularly striking, given there appears to be virtually no spatial clustering of haplotypes (Fig. 2). Female philopatry and relatedness among social groups would explain the pattern in the form of learning (e.g., the majority of white-tailed deer fawns follow their mother's migration route; Nelson 1998); however, our model accounted for such effects through the range covariates (i.e., if daughters were following their mother's migration path they would also share a winter and summer range), and the diversity of haplotypes suggests many different matrilines. In addition, upon examination of individual migratory routes, we found only two deer that shared an identical route. An analysis including males could test this hypothesis (*sensu* Nielsen et al. 2013) or at least be viewed as an independent replicate as males are more prone to disperse (Nelson 1993).

Interestingly, Colorado represents a confluence of several different refugial lineages (Latch et al. 2009), with recolonization routes and so-called hybrid hot spot clusters falling directly in northwestern Colorado (Swenson and Howard 2005). We hypothesize that the mtDNA effect we documented is either: (i) reflective of different refugial histories and biogeography of the mtDNA lineages (Latch et al. 2009), where for example, mule deer originating in northern regions would have locally adapted phenotypes and distinct haplotypes linked to earlier migration times than those from the south (a carryover effect); or (ii) due to differences in energetics related to mtDNA, where, for example, Toews et al. (2014) showed that mitochondrial introgression (where different haplotypes had different energetic outputs) was responsible for differing migratory behavior in a warbler transition zone.

Monteith et al. (2011a) and Lendrum et al. (2013) showed that spring migration timing is closely linked to plant phenology, as deer aim to arrive on their summer range close in time to the peak of plant productivity. Spring arrival dates are more likely to follow plant phenology on individual deer summer ranges, whereas fall migration is linked to weather (temperature and snow on summer range) and individual characteristics such as age and condition. Monteith et al. (2011a) suggested that prime age individuals in the best condition can adopt a strategy by which they stay on summer range for longer time periods to consume higher quality vegetation in spite of the potential for being caught in adverse weather, while poorer quality individuals cannot take on such risks. The individual characteristic hypothesis of Monteith et al. (2011b) provides support for the energetics scenario (ii above), whereby individuals with certain haplotypes might be better suited for taking on the risks associated with remaining on summer range later in the season due to associated differences in energetics.

Fine-scale natal dispersal has been shown to have a heritable basis in albatross (Charmantier et al. 2011), and genotype–phenotype associations are thought to be important next steps in migration studies (Liedvogel et al. 2011). For the carryover effect to be true, the mtDNA lineages must reflect nuclear differences that (at least partially) encode for differences in migratory behavior or have a physiological effect. While our results cannot tease apart a specific nuclear or mitochondrial effect, given the mtDNA migration effect shown in warblers (Toews et al. 2014), we think this is worth following up on using both biochemical modeling and genome-wide scans (i.e., with mtDNA haplotype as the response measure or interaction term). Importantly, recent development in the western United States has raised concerns over the sustainability of mule deer migratory routes (Sawyer et al. 2005, 2009), and under climate change, there is the potential for trophic mismatch for migratory species, whereby migrations occur asynchronously with plant phenology (Post et al. 2008). Monteith et al. (2011b suggested that plasticity in mule deer migration might allow the species to avoid such mismatches; however, if there is a genetic basis for the variability in migration among individuals, there may be less plasticity and more natural selection at work (Nelson 1998). Mitochondrial introgression with white-tailed deer is likely to be unidirectional (Carr et al. 1986), which could jeopardize the adaptive potential if hybridizations increase. However, we note that there is no evidence of white-tailed deer presence in our study area, and thus, hybridization is not a concern at this point. The potential for loss of migratory routes to development combined with climate change and hybridization highlight the importance of maintaining the existing genetic variability in diverse migratory phenotypes.

### Genetic–condition correlations

Fat is an important determinant of fitness for mule deer (Bender et al. 2007; Johnstone-Yellin et al. 2009; Tollefson et al. 2010). We identified two genetic markers as having relationships with fat, although the relationships were antagonistic (i.e., one had a positive relationship with fat and the other negative). Similar results have been seen in studies of both the Kentish plover (*Charadrius alexandrinus* L.; Kupper et al. 2010) and the blue tit (*Parus caeruleus* L.; Olano-Marin et al. 2011). With the contrasting signals of the two markers, interpretations of what these relationships represent become muddled. Olano-Marin et al. (2011) viewed the negative correlation as evidence for direct effects of the neutral loci, with the positive correlation due to inbreeding. Inbreeding in our study area is not supported by the *F*_IS_ values and difficult to imagine given the population size and deer ecology.

Based on the evidence for a mixing of different mitochondrial lineages and effect sizes, the negative relationship to body fat of RT30 (0.99 probability and nearly double the effect size as all other loci) is the most likely to be genuine. However, given the concern over spurious HFCs, we must still consider the possibility of Type I errors (i.e., false positives). The potential for type I errors is of particular concern when detecting local effects and examining multiple models (Szulkin et al. 2010). In light of this concern, we highlight three points of support for the recorded relationship. First, the effect sizes of the significant coefficients were substantially greater than those of the other loci (Fig. 3). Second, we refit all models that had significant coefficients, but with a strong mean 0 multivariate normal prior on the coefficients. This approach shrinks all estimates toward 0, acting as a penalty and reducing the number of significant covariates (Gelman et al. 2012). In the case of the SLH – fat correlation, all significant results (probability of an effect > 0.95) remained. Lastly, the proximity of a locus in question relative to genes of known effect can be taken as supportive evidence for understanding single-locus HFCs (Von Hardenberg et al. 2007; Kupper et al. 2010). Slate et al. (2002) observed considerable synteny in ruminants, and more than half of the microsatellites used in their deer linkage map had been used for the same purposes in cow and sheep. When we screened RT30 against the annotated cow genome (using blast), both primers colocalized with 100% identity to a region with the closest known gene being that of TBC1D1. Interestingly, this gene regulates cell growth and differentiation and has been shown to influence fat metabolism in mice and humans (Stone et al. 2006; Chadt et al. 2008). Given the combination of divergent mtDNA lineages in our study area and panmixia (*k* = 1), a slight disruption of co-adapted alleles that are linked to fat metabolism could explain the negative correlation between this locus and fat (we emphasize these results represent a small effect as body fat was predicted to decrease body fat by <0.2% in the model). This is predicted to outcome when locally adapted lineages mix, and it has been recently suggested for grizzly bears in an area where they are subject to large-scale human-assisted migration (Shafer et al. 2014).

While the above lines of evidence offer support to the effect of RT30 on fat being genuine, given the small number of loci examined, we must remain skeptical about this relationship. Rather, we present these findings as noteworthy and in need of confirmation by studies with larger samples and with genomic methods.

### Conclusions and evolutionary applications

We have shown fine-scale relationships between genetic variation and phenotypic traits in mule deer that have not been found in previous work on this species. Our study identified fine-scale genetic correlates to both migration timing and body fat that are likely overlooked (and probably unexpected) in this species. These results have potential management implications for mule deer, which are under substantial human pressure from a multitude of stressors (Sawyer et al. 2006). The genetic polymorphisms in this population that are linked to phenotypic traits related to phenology and metabolic variation could prove important in the face of climate change and other anthropogenic stressors that are likely to affect both optimal timing of migration and the role of fat stores in survival and reproduction. Monitoring hybridization with white-tailed deer should also be considered with respect to the mtDNA effect, as introgression is likely to go from white-tailed to mule deer (Carr et al. 1986) and could alter the adaptive potential. Efforts should be made to better characterize additional drivers behind this phenotypic and genetic variation in an effort to maintain a diversity of phenotypes that might best be able to adapt to novel conditions. Screening for similar associations in more imperiled deer populations (and cervid species) may help shed light on local population dynamics and better inform management decisions.

## Data archiving statement

Data for this study are available from GenBank Accession Numbers KM061069–KM061151 and from the Dryad Digital Repository: http://doi.org/10.5061/dryad.3vc1b.

## Supporting Information

**Appendix S1**. PCR conditions.

**Appendix S2.** Microsatellite diversity statistics **Table 1**. Microsatellite loci, number of individuals genotyped (*N*), number of alleles present at each loci (Na), observed heterozygosity (Ho), expected heterozygosity (He), and fixation index (*F*).

**Appendix S3.** Bayesian model formulations.

**Appendix S4.** Phylogenetic trees.

**Appendix S5.** Supplemental results. **Table 1**. Identification (ID) numbers, ages, study area, and whether individual was captured in December, March or both for mule deer captured in the Piceance basin of Colorado. **Table 2**. DIC values for multi-level linear regression models on mule deer body mass, and multi-level beta regression models on mule deer body fat relative to MLH (multi-locus heterozygosity) or SLH (single-locus heterozygosity). **Table 3**. Covariates, median coefficient (coeff.) values, and the probability (prob.) of either a negative or positive effect of the covariate on mule deer body mass and body fat estimated from multi-level linear or beta regression respectively. **Table 4**. Covariates, median coefficient (coeff.) values, and the probability (prob.) of either a negative or positive effect of the covariate on mule deer Spring migration termination date estimated from negative binomial regression model from mule deer captured in the Piceance basin, Colorado. **Table 5**. Covariates, median coefficient (coeff.) values, and the probability (prob.) of either a negative or positive effect of the covariate on mule deer Spring migration initiation date estimated from negative binomial regression model from mule deer captured in the Piceance basin, Colorado. **Table 6**. Covariates, median coefficient (coeff.) values, and the probability (prob.) of either a negative or positive effect of the covariate on mule deer Fall migration initiation date estimated from negative binomial regression model from mule deer captured in the Piceance basin, Colorado. **Figure 1**. Fitted values versus residuals from negative binomial model fit to migration timing of mule deer in the Piceance basin of Colorado. **Figure 2**. Fitted values versus residuals from negative binomial model fit to migration timing of mule deer in the Piceance basin of Colorado. The residuals were calculated from the model including mtDNA clades determined from the neighbor joining analysis. **Figure 3**. Fitted values versus residuals from hierarchical beta regression fit to percent body fat of mule deer in the Piceance basin of Colorado.
