# Supplementary material for: Fine-scale genetic correlates to condition and migration in a wild cervid
Source: Evol Appl. 2014 Aug 28;7(8):937–48. doi: 10.1111/eva.12189 (PMC4211723; doi:10.1111/eva.12189)
Supplement: Supplementary file 1 — Appendix S1. PCR conditions. [file eva0007-0937-sd1.docx]

**APPENDIX S1: PCR conditions**

The multiplex microsatellite reaction consisted of 25 ng DNA, 5 μL 2× Qiagen Multiplex mix, 2 μL primer mix, and 0.5 μL distilled water. The 10 μl single reactions contained 0.8 μL of MgCl_2_ (20 mM), 1 μL 10× PCR buffer, 2 μL of dNTPs (0.2 mM each), a 20× primer mix diluted to between 0.24 and 0.34 μL each, 0.08 μL of Taq (0.5 units), 1 μL of DNA template (~10 ng) and Milli-Q water. One primer per pair was fluorescently labelled. The multiplex PCR parameters followed Cullingham et al. (2011) and the single-PCRs began with an initial 3-minute denaturation at 95°C, followed by 38 cycles of 30 seconds denaturation at 94°C, 90 seconds annealing at 49°C, and 30 seconds extension at 72°C. The microsatellite amplicons were loaded on an ABI 3730 DNA sequencer (Applied Biosystems, Foster City, CA, USA) with a GS500LIZ size standard (Applied Biosystems). Microsatellite alleles were scored using GENEMAPPER version 4.0 (Applied Biosystems) and deviations from Hardy-Weinberg equilibrium (HWE) were tested using the exact test (Guo and Thompson 1992) implemented in Genepop v.4.0 (Rousset 2008)and FSTAT v.2.9.3 (Goudet 1995)was used to test for linkage disequilibrium.

The mitochondrial control region was amplified in a 25 μl PCR reaction containing ~50 ng of template DNA, 0.2 mM each dNTP, 1× PCR buffer, 0.2 μM each primer, 1.6 mM MgCl_2_, 0.1 U Taq DNA polymerase, and Milli-Q water. The PCR profile was as follows: hot-start followed by an initial 2-minute denaturation at 94°C, followed by 38 cycles of 30 seconds denaturation at 94°C, 58°C, 72°C. The run concluded after 5 minutes at 72°C. PCR success was determined from gel electrophoresis. PCR product (10 μl) was treated with 5 μl of ExoSAP (USB Corporation, OH, USA) and incubated at 37°C for 15 minutes followed by 80°C for 15 minutes. The ExoSAP treated PCR product was used in a sequencing reaction. Amplicons were directly sequenced in both directions using a Big Dye Terminator Kit (Applied Biosystems, Foster City, CA) and generated on an ABI 3730. Sequences were aligned using the ClustalW algorithm (Thompson et al. 1994).
