# Supplementary material for: Fine-scale genetic correlates to condition and migration in a wild cervid
Source: Evol Appl. 2014 Aug 28;7(8):937–48. doi: 10.1111/eva.12189 (PMC4211723; doi:10.1111/eva.12189)
Supplement: Supplementary file 2 [file eva0007-0937-sd2.docx]

**APPENDIX S2: Bayesian model formulations**

Multi-level linear regression model:

$${\log(mass}_{ij})\sim Normal\left( \mu_{ij}, \sigma^{2} \right)$$

$$\mu_{ij}=\alpha_{i}+\boldsymbol{x}_{\boldsymbol{ij}}^{\boldsymbol{'}}\boldsymbol{\beta}$$

$$\alpha_{i}\sim Normal(\mu_{\alpha}, \sigma_{\alpha}^{2})$$

$$\mu_{\alpha}\sim Normal(0, 300)$$

$$\sigma_{\alpha}=\frac{1}{\sqrt{\tau_{\alpha}}}$$

$$\tau_{\alpha}\sim gamma(0.001, 0.001)$$

$$\boldsymbol{\beta}\boldsymbol{\sim}Normal(\boldsymbol{0}, 10 000I)$$

$$\sigma=\frac{1}{\sqrt{\tau}}$$

$$\tau\sim gamma(0.001, 0.001)$$

Multi-level beta regression model:

$${fat}_{ij}\sim beta\left( a_{ij}, b_{ij} \right)$$

$$a_{ij}=\mu_{ij}\times\phi$$

$$b_{ij}=\left( 1-\mu_{ij} \right)\phi$$

$${logit(\mu}_{ij})=\alpha_{i}+\boldsymbol{x}_{\boldsymbol{ij}}^{\boldsymbol{'}}\boldsymbol{\beta}$$

$$\alpha_{i}\sim Normal(\mu_{\alpha}, \sigma_{\alpha}^{2})$$

$$\mu_{\alpha}\sim Normal(0, 300)$$

$$\sigma_{\alpha}=\frac{1}{\sqrt{\tau_{\alpha}}}$$

$$\tau_{\alpha}\sim gamma(0.001, 0.001)$$

$$\boldsymbol{\beta}\boldsymbol{\sim}Normal(\boldsymbol{0}, 10 000I)$$

$$\phi\sim gamma(0.0001, 0.0001)$$

Negative binomial regression model:

$${days}_{j}\sim Negative binomial\left( p_{j}, r \right)$$

$$p_{j}=\frac{r}{r+\lambda_{j}}$$

$$\log\left( \lambda_{j} \right)=\boldsymbol{x}_{\boldsymbol{j}}^{\boldsymbol{'}}\boldsymbol{\beta}$$

$$\boldsymbol{\beta}\boldsymbol{\sim}Normal(\boldsymbol{0}, 10 000I)$$

$$r\sim Uniform(0,100)$$

In all models, $i$ indexes the individual, $j$ indexes the observation, $\boldsymbol{x}_{\boldsymbol{ij}}$ is vector of covariates for the $i^{th}$ individual and $j^{th}$ observation with corresponding vector of coefficients, $\boldsymbol{\beta}$. The above formulations include priors, which were formulated to be diffuse.

**Specifics of model runs**

For all models 2 Markov Chain Monte Carlo (MCMC) runs were used, with initial values that were expected to be overdispersed relative to the posterior distribution. Convergence to the posterior distribution was assessed using the Gelman-Rubin diagnostic (Gelman and Rubin 1992), and by examining trace plots of the MCMCs. We ran the negative binomial models of migration timing for 400,000 iterations, discarding the first 100,000 as burn-in. We ran the MLH models for condition for 300,000 iterations, discarding the first 100,000 as burn-in. We ran the SLH models for condition for 400,000 iterations discarding the first 100,000 as burn-in. Once convergence was reached we calculated the median of the posterior distributions for all coefficients, as well as the probability that each coefficient was above and below 0.

**Literature cited**

Gelman, A. and D. B. Rubin. 1992. Inference from iterative simulation using multiple sequences. Statistical Science **7**:457-511.
