# Supplementary material for: Fine-scale genetic correlates to condition and migration in a wild cervid
Source: Evol Appl. 2014 Aug 28;7(8):937–48. doi: 10.1111/eva.12189 (PMC4211723; doi:10.1111/eva.12189)
Supplement: Supplementary file 3 — Appendix S3. Bayesian model formulations. [file eva0007-0937-sd3.docx]

| Locus | N | Na | Ho | He | F |
| --- | --- | --- | --- | --- | --- |
| INRA011a | 134 | 6.000 | 0.500 | 0.489 | -0.023 |
| RT30a | 132 | 14.000 | 0.788 | 0.794 | 0.008 |
| BBJ2a | 134 | 8.000 | 0.739 | 0.781 | 0.054 |
| Ka | 135 | 5.000 | 0.748 | 0.725 | -0.032 |
| BL25a | 133 | 6.000 | 0.767 | 0.706 | -0.086 |
| BM6438a | 134 | 10.000 | 0.784 | 0.732 | -0.071 |
| BM848a | 135 | 9.000 | 0.748 | 0.755 | 0.010 |
| RT7a | 133 | 8.000 | 0.827 | 0.786 | -0.052 |
| Na | 135 | 12.000 | 0.852 | 0.881 | 0.033 |
| ETH152a | 134 | 10.000 | 0.791 | 0.803 | 0.015 |
| BM6506a | 135 | 5.000 | 0.741 | 0.701 | -0.056 |
| Pa | 132 | 7.000 | 0.538 | 0.550 | 0.021 |
| Da | 132 | 6.000 | 0.462 | 0.463 | 0.001 |
| BM4107a | 134 | 11.000 | 0.828 | 0.838 | 0.011 |
| RT5a | 134 | 10.000 | 0.836 | 0.777 | -0.075 |
| OCAMa | 129 | 8.000 | 0.628 | 0.558 | -0.125 |
| Ra | 131 | 6.000 | 0.634 | 0.619 | -0.024 |
| Mean | 133.294 | 8.294 | 0.718 | 0.703 | -0.023 |
| SE | 0.400 | 0.629 | 0.029 | 0.030 | 0.012 |

**APPENDIX S3: Microsatellite diversity statistics**

Table 1. Microsatellite loci, number of individuals genotyped (N), number of alleles present at each loci (Na), observed heterozygosity (Ho), expected heterozygosity (He), and fixation index (F).
