# Supplementary figures and images for: Fine-scale genetic correlates to condition and migration in a wild cervid
Source: Evol Appl. 2014 Aug 28;7(8):937–48. doi: 10.1111/eva.12189 (PMC4211723; doi:10.1111/eva.12189)

## Appendix D: Phylogenetic trees

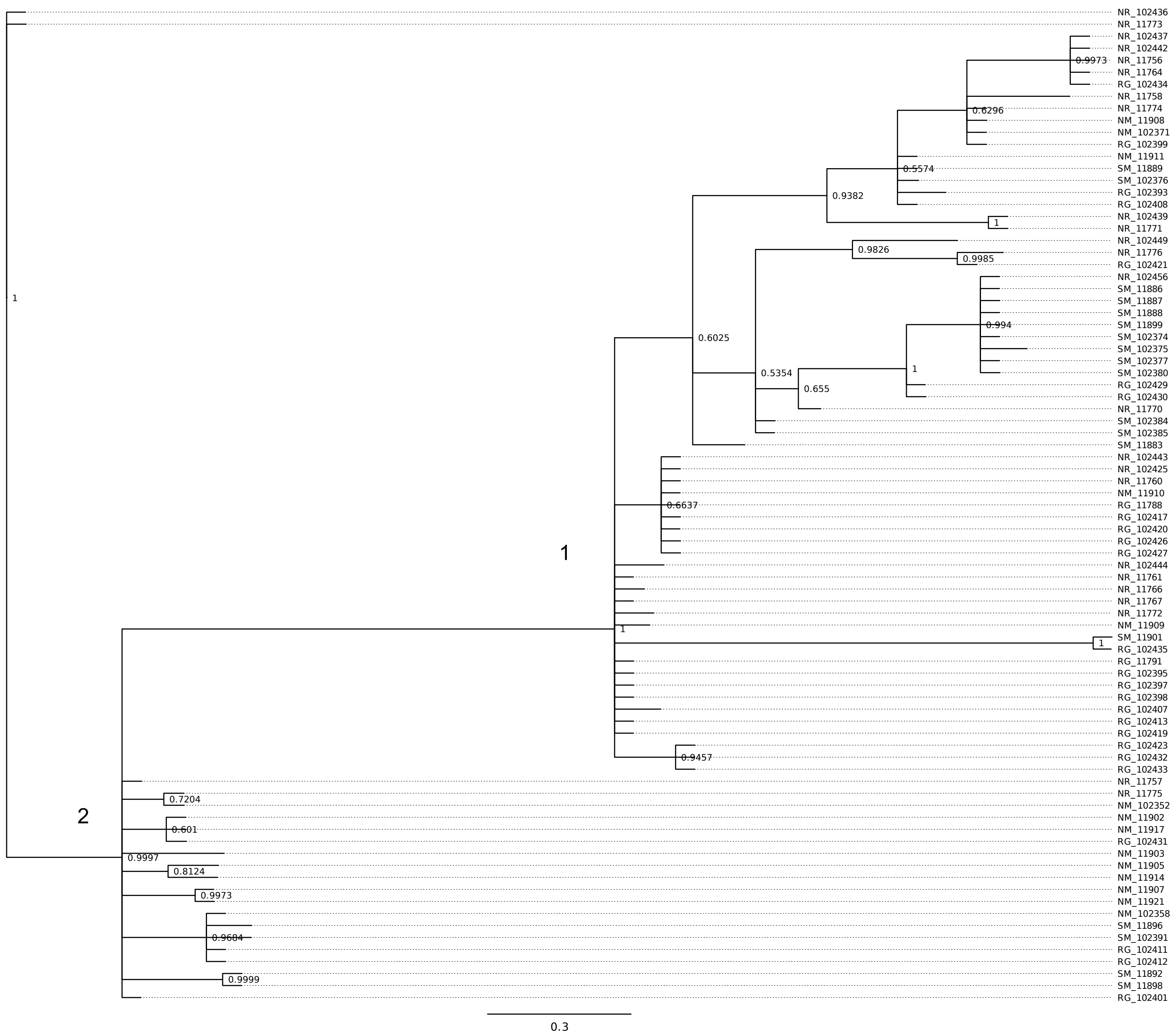

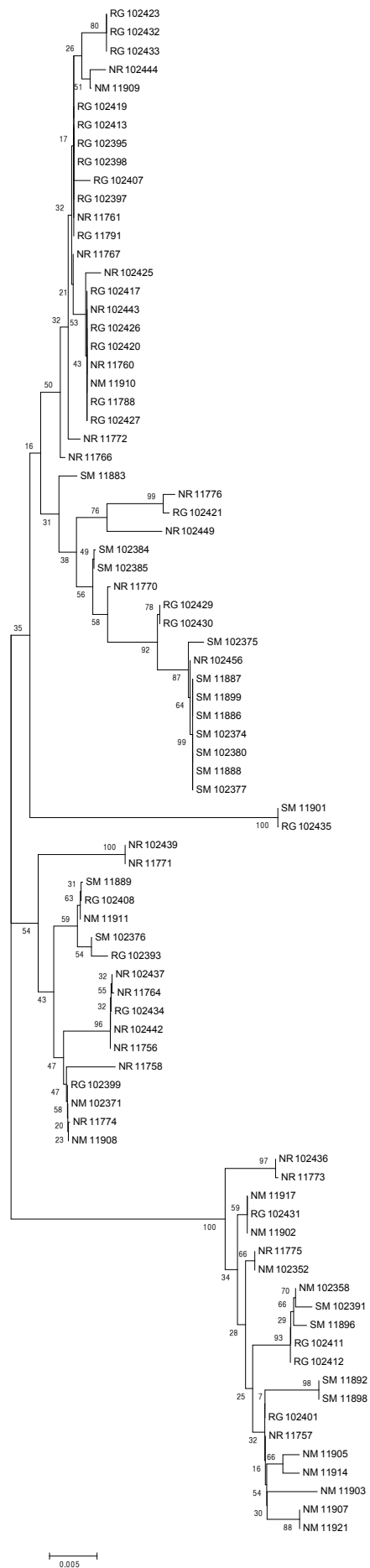

2

3

1

Supplement: Supplementary file 4 — Appendix S4. Phylogenetic trees. [file eva0007-0937-sd4.pdf]
