# Supplementary material for: Fine-scale genetic correlates to condition and migration in a wild cervid
Source: Evol Appl. 2014 Aug 28;7(8):937–48. doi: 10.1111/eva.12189 (PMC4211723; doi:10.1111/eva.12189)
Supplement: Supplementary file 5 [file eva0007-0937-sd5.docx]

**APPENDIX S5: supplemental results**

Table 1. Identification (ID) numbers, ages, study area, and whether individual was captured in December, March or both for mule deer captured in the Piceance basin of Colorado.

| ID number | Age | Study area | Capture period |
| --- | --- | --- | --- |
| 11890 | 4.5 | SM | December |
| 102352 | 5.5 | NM | December |
| 102353 | 9.5 | NM | December |
| 102354 | 5.5 | NM | December |
| 102357 | 10.5 | NM | December |
| 102358 | 2.5 | NM | December |
| 102363 | 5.5 | NM | December |
| 102365 | 5.5 | NM | December |
| 102368 | 3.5 | NM | December |
| 102370 | 3.5 | NM | December |
| 102371 | 10.5 | NM | December |
| 102374 | 8.5 | SM | December |
| 102375 | 2.5 | SM | December |
| 102376 | 10.5 | SM | December |
| 102377 | 5.5 | SM | December |
| 102380 | 2.5 | SM | December |
| 102384 | 7.5 | SM | December |
| 102385 | 1.5 | SM | December |
| 102386 | 4.5 | SM | December |
| 102390 | 7.5 | SM | December |
| 102391 | 4.5 | SM | December |
| 102392 | 4.5 | RG | December |
| 102396 | 7.5 | RG | December |
| 102398 | 3.5 | RG | December |
| 102407 | 9.5 | RG | December |
| 102408 | 6.5 | RG | December |
| 102409 | 4.5 | RG | December |
| 102410 | 1.5 | RG | December |
| 102411 | 4.5 | RG | December |
| 102412 | 7.5 | RG | December |
| 102413 | 4.5 | RG | December |
| 102416 | 3.5 | RG | December |
| 102417 | 6.5 | RG | December |
| 102418 | 3.5 | RG | December |
| 102419 | 5.5 | RG | December |
| 102420 | 4.5 | RG | December |
| 102421 | 10.5 | RG | December |
| 102423 | 2.5 | RG | December |
| 102426 | 7.5 | RG | December |
| 102427 | 9.5 | RG | December |
| 102429 | 7.5 | RG | December |
| 102430 | 9.5 | RG | December |
| 102431 | 10.5 | RG | December |
| 102434 | 5.5 | RG | December |
| 102435 | 4.5 | RG | December |
| 102436 | 7.5 | NR | December |
| 102442 | 7.5 | NR | December |
| 102443 | 10.5 | NR | December |
| 102444 | 4.5 | NR | December |
| 102448 | 6.5 | NR | December |
| 102449 | 3.5 | NR | December |
| 102452 | 2.5 | NR | December |
| 102455 | 3.5 | NR | December |
| 102456 | 4.5 | NR | December |
| 102457 | 8.5 | NR | December |
| 11758 | 9.5 | NR | Both |
| 11760 | 5.5 | NR | Both |
| 11761 | 2.5 | NR | Both |
| 11767 | 4.5 | NR | Both |
| 11768 | 6.5 | NR | Both |
| 11769 | 8.5 | NR | Both |
| 11771 | 3.5 | NR | Both |
| 11772 | 8.5 | NR | Both |
| 11778 | 5.5 | RG | Both |
| 11779 | 5.5 | RG | Both |
| 11780 | 4.5 | RG | Both |
| 11781 | 4.5 | RG | Both |
| 11782 | 6.5 | RG | Both |
| 11783 | 4.5 | RG | Both |
| 11784 | 5.5 | RG | Both |
| 11787 | 8.5 | RG | Both |
| 11788 | 3.5 | RG | Both |
| 11789 | 6.5 | RG | Both |
| 11791 | 4.5 | RG | Both |
| 11792 | 3.5 | RG | Both |
| 11885 | 2.5 | SM | Both |
| 11886 | 5.5 | SM | Both |
| 11887 | 6.5 | SM | Both |
| 11888 | 3.5 | SM | Both |
| 11889 | 5.5 | SM | Both |
| 11891 | 10.5 | SM | Both |
| 11894 | 10.5 | SM | Both |
| 11899 | 4.5 | SM | Both |
| 11903 | 3.5 | NM | Both |
| 11905 | 3.5 | NM | Both |
| 11906 | 2.5 | NM | Both |
| 11908 | 3.5 | NM | Both |
| 11910 | 8.5 | NM | Both |
| 11911 | 4.5 | NM | Both |
| 11913 | 6.5 | NM | Both |
| 11916 | 10.5 | NM | Both |
| 11919 | 2.5 | NM | Both |
| 11920 | 2.5 | NM | Both |
| 102393 | 6.5 | RG | Both |
| 102395 | 8.5 | RG | Both |
| 102397 | 6.5 | RG | Both |
| 102399 | 4.5 | RG | Both |
| 102401 | 3.5 | RG | Both |
| 102432 | 3.5 | RG | Both |
| 102433 | 9.5 | RG | Both |
| 102437 | 5.5 | NR | Both |
| 11756 | 7.5 | NR | March |
| 11759 | 7.5 | NR | March |
| 11762 | 1.5 | NR | March |
| 11764 | 7.5 | NR | March |
| 11765 | 2.5 | NR | Both |
| 11766 | 5.5 | NR | Both |
| 11770 | 6.5 | NR | March |
| 11773 | 6.5 | NR | March |
| 11774 | 5.5 | NR | March |
| 11775 | 3.5 | NR | March |
| 11776 | 2.5 | NR | March |
| 11777 | 11.5 | RG | March |
| 11882 | 5.5 | SM | March |
| 11883 | 7.5 | SM | March |
| 11884 | 8.5 | SM | March |
| 11892 | 4.5 | SM | March |
| 11893 | 8.5 | SM | March |
| 11895 | 10.5 | SM | March |
| 11896 | 4.5 | SM | March |
| 11897 | 3.5 | SM | March |
| 11898 | 6.5 | SM | March |
| 11900 | 7.5 | SM | March |
| 11901 | 3.5 | SM | March |
| 11902 | 7.5 | NM | March |
| 11904 | 3.5 | NM | March |
| 11907 | 7.5 | NM | March |
| 11909 | 4.5 | NM | March |
| 11912 | 6.5 | NM | March |
| 11914 | 4.5 | NM | March |
| 11915 | 8.5 | NM | March |
| 11917 | 4.5 | NM | March |
| 11918 | 8.5 | NM | March |
| 11921 | 6.5 | NM | March |

Table 2. DIC values for multi-level linear regression models on mule deer body mass, and multi-level beta regression models on mule deer body fat relative to MLH (multi-locus heterozygosity) or SLH (single-locus heterozygosity). Body mass and fat were calculated from deer captured via helicopter net-gunning on their winter range.

| Dependent variable | Model | Model structure | DIC |
| --- | --- | --- | --- |
| Mass | M1 | MLH + Age + March Capture + Study area | -338.9 |
| Mass | M2 | MLH + Age + Age^2^ + March Capture + Study area | -339.2 |
| Mass | M3 | SLH^*^ + Age + March Capture + Study area | -334.5 |
| Mass | M4 | SLH^*^ + Age + Age^2^ + March Capture + Study area | -334.6 |
| Fat | F1 | MLH + Age + March Capture + Study area | -695.9 |
| Fat | F2 | MLH + Age + Age^2^ + March Capture + Study area | -695.8 |
| Fat | F3 | SLH^*^ + Age + March Capture + Study area | -691.6 |
| Fat | F4 | SLH^*^ + Age + Age^2^ + March Capture + Study area | -690.9 |

^*^SLH indicates a set of 17 dummy variables indicating if the individual was heterozygous (1) or not (0) at a specific locus.

Table 3. Covariates, median coefficient (coeff.) values, and the probability (prob.) of either a negative or positive effect of the covariate on mule deer body mass and body fat estimated from multi-level linear or beta regression respectively. Models presented are lowest DIC models for both MLH and SLH models of mass and body fat.

| Mass model M2 |  |  |  |
| --- | --- | --- | --- |
| Covariate | Median coeff. value | Prob. coeff. is negative | Prob. coeff. positive |
| MLH | 0.117 | 0.24 | 0.76 |
| Age | 0.036 | 0.03 | 0.97 |
| Age^2^ | -0.021 | 0.91 | 0.09 |
| March Capture | -0.1 | 1 | 0 |
| NR^*^ | -0.014 | 0.61 | 0.39 |
| RG^†^ | -0.02 | 0.67 | 0.33 |
| SM^‡^ | -0.007 | 0.56 | 0.44 |
|  |  |  |  |
| Mass model M4 |  |  |  |
| Covariate | Median coeff. value | Prob. coeff. is negative | Prob. coeff. positive |
| Age | 0.033 | 0.06 | 0.94 |
| Age^2^ | -0.021 | 0.89 | 0.11 |
| March Capture | -0.100 | 1 | 0 |
| NR^*^ | -0.014 | 0.60 | 0.40 |
| RG^†^ | -0.027 | 0.69 | 0.31 |
| SM^‡^ | -0.027 | 0.69 | 0.31 |
| INRA011 | -0.024 | 0.73 | 0.27 |
| RT30 | -0.060 | 0.90 | 0.10 |
| BBJ | 0.024 | 0.30 | 0.70 |
| K | 0.036 | 0.20 | 0.80 |
| BL25 | -0.004 | 0.53 | 0.47 |
| BM6438 | 0.010 | 0.42 | 0.58 |
| BM848 | 0.028 | 0.27 | 0.73 |
| RT7 | 0.030 | 0.29 | 0.71 |
| N | 0.085 | 0.06 | 0.94 |
| ETH152 | -0.034 | 0.77 | 0.23 |
| BM6506 | 0.036 | 0.21 | 0.79 |
| P | 0.002 | 0.48 | 0.52 |
| D | 0.010 | 0.40 | 0.60 |
| BM4107 | 0.044 | 0.21 | 0.79 |
| RT5 | 0.032 | 0.27 | 0.73 |
| OCAM | 0.027 | 0.25 | 0.75 |
| R | -0.038 | 0.82 | 0.18 |
|  |  |  |  |
| Fat model F1 |  |  |  |
| Covariate | Median coeff. value | Prob. coeff. is negative | Prob. coeff. positive |
| MLH | 0.094 | 0.39 | 0.61 |
| Age | -0.043 | 0.87 | 0.13 |
| March Capture | -0.515 | 1 | 0 |
| NR^*^ | -0.078 | 0.74 | 0.26 |
| RG^†^ | -0.026 | 0.60 | 0.4 |
| SM^‡^ | 0.041 | 0.36 | 0.64 |
|  |  |  |  |
| Fat model F3 |  |  |  |
| Covariate | Median coeff. value | Prob. coeff. is negative | Prob. coeff. positive |
| Age | -0.051 | 0.89 | 0.11 |
| March Capture | -0.518 | 1 | 0 |
| NR^*^ | -0.104 | 0.80 | 0.20 |
| RG^†^ | -0.105 | 0.82 | 0.18 |
| SM^‡^ | -0.059 | 0.69 | 0.31 |
| INRA011 | -0.127 | 0.93 | 0.07 |
| RT30 | -0.240 | 0.99 | 0.01 |
| BBJ | 0.077 | 0.22 | 0.78 |
| K | -0.034 | 0.65 | 0.35 |
| BL25 | 0.074 | 0.27 | 0.73 |
| BM6438 | -0.001 | 0.50 | 0.50 |
| BM848 | -0.108 | 0.87 | 0.13 |
| RT7 | -0.078 | 0.72 | 0.28 |
| N | 0.087 | 0.22 | 0.78 |
| ETH152 | -0.004 | 0.52 | 0.48 |
| BM6506 | 0.024 | 0.40 | 0.60 |
| P | 0.175 | 0.04 | 0.96 |
| D | 0.092 | 0.13 | 0.87 |
| BM4107 | 0.053 | 0.32 | 0.68 |
| RT5 | 0.145 | 0.13 | 0.87 |
| OCAM | 0.020 | 0.41 | 0.59 |
| R | -0.076 | 0.81 | 0.19 |

^*^Indicates deer captured in the NR study area, with NM as the reference category

^†^Indicates deer captured in the RG study area, with NM as the reference category

^‡^Indicates deer captured in the SM study area, with NM as the reference category

Table 4. Covariates, median coefficient (coeff.) values, and the probability (prob.) of either a negative or positive effect of the covariate on mule deer Spring migration termination date estimated from negative binomial regression model from mule deer captured in the Piceance basin, Colorado.

| Neighbor joining clades |  |  |  |
| --- | --- | --- | --- |
| Covariate | Median coeff. value | Prob. coeff. is negative | Prob. coeff. positive |
| Intercept | 3.48 | 0 | 1 |
| Age | 0.01 | 0.45 | 0.55 |
| NR^*^ | -0.23 | 0.93 | 0.07 |
| RG^†^ | -0.01 | 0.52 | 0.48 |
| SM^‡^ | -0.02 | 0.56 | 0.44 |
| mtDNA cluster 2^§^ | 0.04 | 0.38 | 0.62 |
| mtDNA cluster 3^§^ | 0.04 | 0.39 | 0.61 |
|  |  |  |  |
| Bayesian clades |  |  |  |
| Covariate | Median coeff. value | Prob. coeff. is negative | Prob. coeff. positive |
| Intercept | 3.49 | 0 | 1 |
| Age | 0.01 | 0.44 | 0.56 |
| NR^*^ | -0.23 | 0.93 | 0.07 |
| RG^†^ | -0.02 | 0.55 | 0.45 |
| SM^‡^ | -0.03 | 0.58 | 0.42 |
| mtDNA cluster 2^§^ | 0.03 | 0.41 | 0.59 |

^*^Indicates deer captured in the NR study area, with NM as the reference category

^†^Indicates deer captured in the RG study area, with NM as the reference category

^‡^Indicates deer captured in the SM study area, with NM as the reference category

^§^mtDNA cluster 1 is the reference category

Table 5. Covariates, median coefficient (coeff.) values, and the probability (prob.) of either a negative or positive effect of the covariate on mule deer Spring migration initiation date estimated from negative binomial regression model from mule deer captured in the Piceance basin, Colorado.

| Neighbor joining clades |  |  |  |
| --- | --- | --- | --- |
| Covariate | Median coeff. value | Prob. coeff. is negative | Prob. coeff. positive |
| Intercept | 3.41 | 0 | 1 |
| Age | -0.03 | 0.7 | 0.3 |
| NR^*^ | -0.67 | 1 | 0 |
| RG^†^ | -0.14 | 0.79 | 0.21 |
| SM^‡^ | -0.08 | 0.67 | 0.33 |
| mtDNA cluster 2^§^ | 0.01 | 0.48 | 0.52 |
| mtDNA 3 | -0.12 | 0.76 | 0.24 |
|  |  |  |  |
| Bayesian clades |  |  |  |
| Covariate | Median coeff. value | Prob. coeff. is negative | Prob. coeff. positive |
| Intercept | 3.36 | 0 | 1 |
| Age | -0.04 | 0.76 | 0.24 |
| NR^*^ | -0.66 | 1 | 0 |
| RG^†^ | -0.11 | 0.74 | 0.26 |
| SM^‡^ | -0.05 | 0.32 | 0.38 |
| mtDNA cluster 2^§^ | 0.04 | 0.38 | 0.62 |

^*^Indicates deer captured in the NR study area, with NM as the reference category

^†^Indicates deer captured in the RG study area, with NM as the reference category

^‡^Indicates deer captured in the SM study area, with NM as the reference category

^§^mtDNA cluster 1 is the reference category

Table 6. Covariates, median coefficient (coeff.) values, and the probability (prob.) of either a negative or positive effect of the covariate on mule deer Fall migration initiation date estimated from negative binomial regression model from mule deer captured in the Piceance basin, Colorado.

| Neighbor joining clades |  |  |  |
| --- | --- | --- | --- |
| Covariate | Median coeff. value | Prob. coeff. is negative | Prob. coeff. positive |
| Intercept | 3.07 | 0 | 1 |
| Age | -0.17 | 0.92 | 0.08 |
| NR^*^ | -0.1 | 0.62 | 0.38 |
| RG^†^ | -0.44 | 0.91 | 0.09 |
| SM^‡^ | -0.78 | 0.98 | 0.02 |
| mtDNA cluster 2^§^ | -0.52 | 0.96 | 0.04 |
| mtDNA cluster 3^§^ | -0.55 | 0.95 | 0.05 |
|  |  |  |  |
| Bayesian clades |  |  |  |
| Covariate | Median coeff. value | Prob. coeff. is negative | Prob. coeff. positive |
| Intercept | 2.84 | 0 | 1 |
| Age | -0.18 | 0.93 | 0.07 |
| NR^*^ | -0.08 | 0.59 | 0.41 |
| RG^†^ | -0.28 | 0.81 | 0.19 |
| SM^‡^ | -0.59 | 0.95 | 0.05 |
| mtDNA cluster 2^§^ | -0.35 | 0.9 | 0.1 |

^*^Indicates deer captured in the NR study area, with NM as the reference category

^†^Indicates deer captured in the RG study area, with NM as the reference category

^‡^Indicates deer captured in the SM study area, with NM as the reference category

^§^mtDNA cluster 1 is the reference category


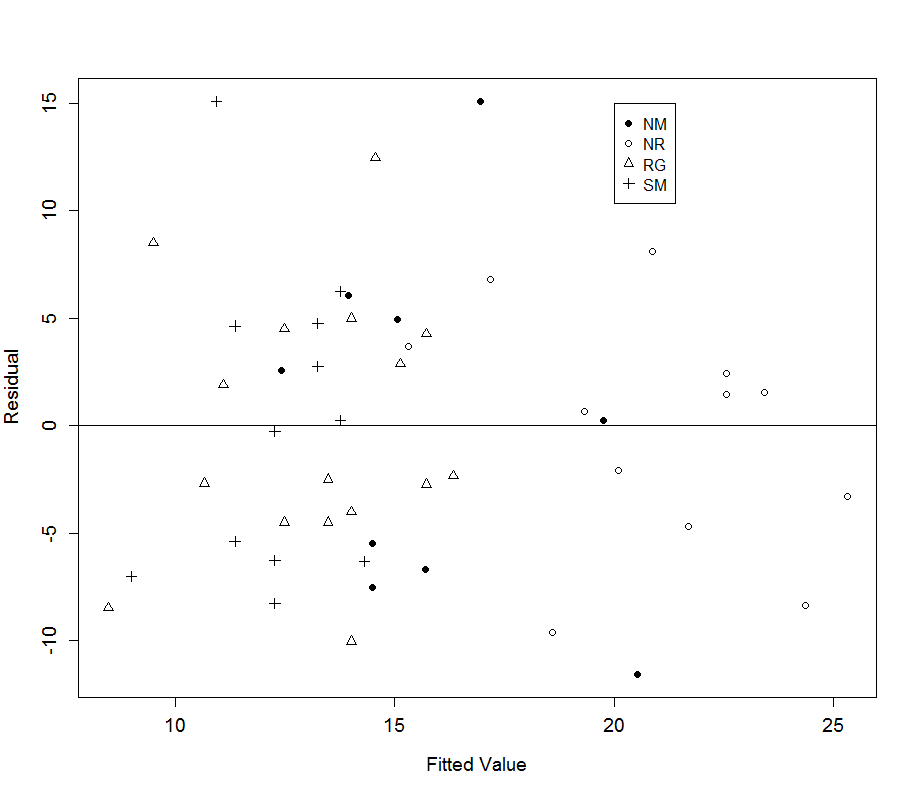


Fig. 1. Fitted values versus residuals from negative binomial model fit to migration timing of mule deer in the Piceance basin of Colorado. The residuals were calculated from the model including mtDNA clades determined from the Bayesian analysis.


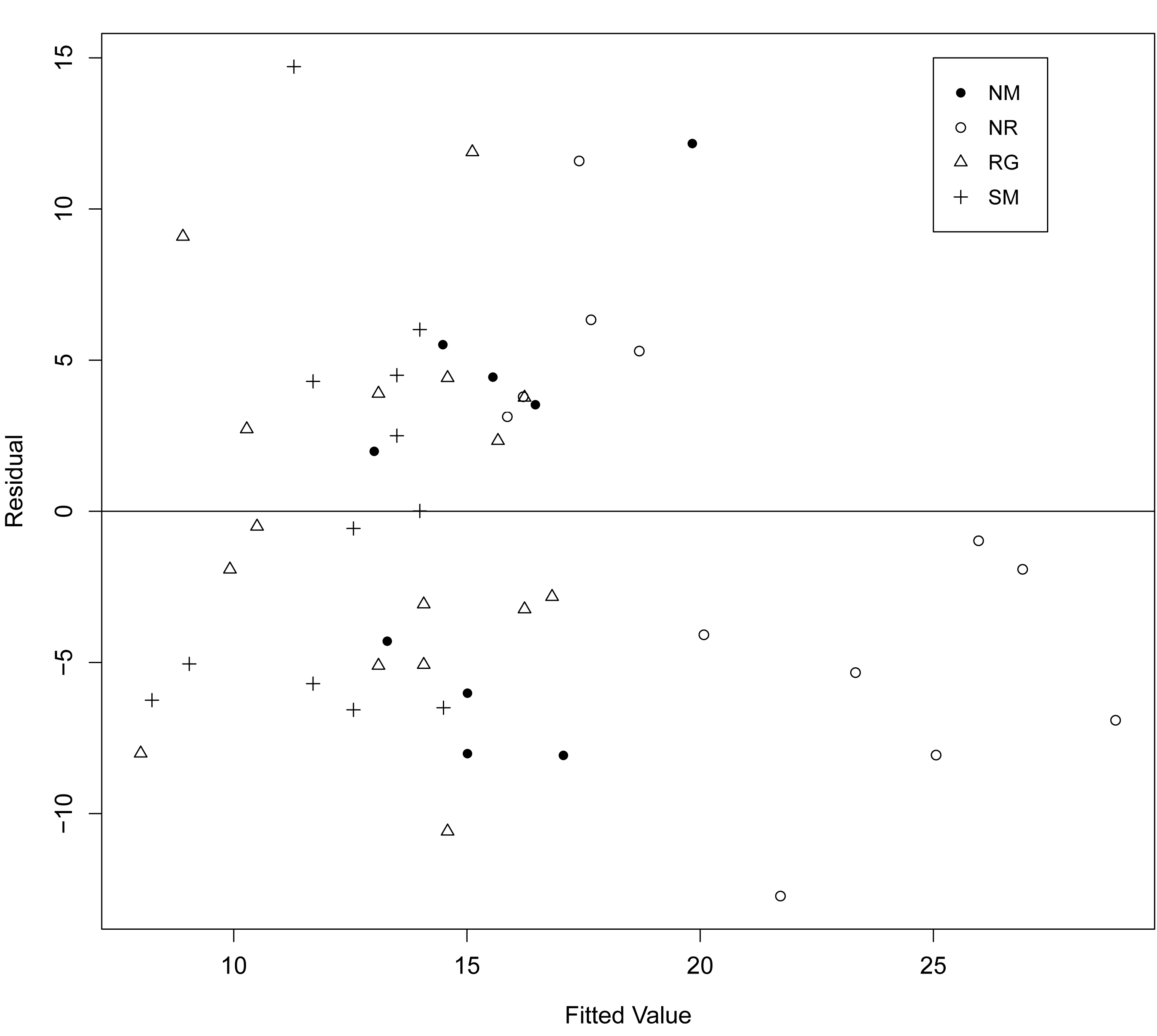


Fig. 2. Fitted values versus residuals from negative binomial model fit to migration timing of mule deer in the Piceance basin of Colorado. The residuals were calculated from the model including mtDNA clades determined from the neighbor joining analysis.


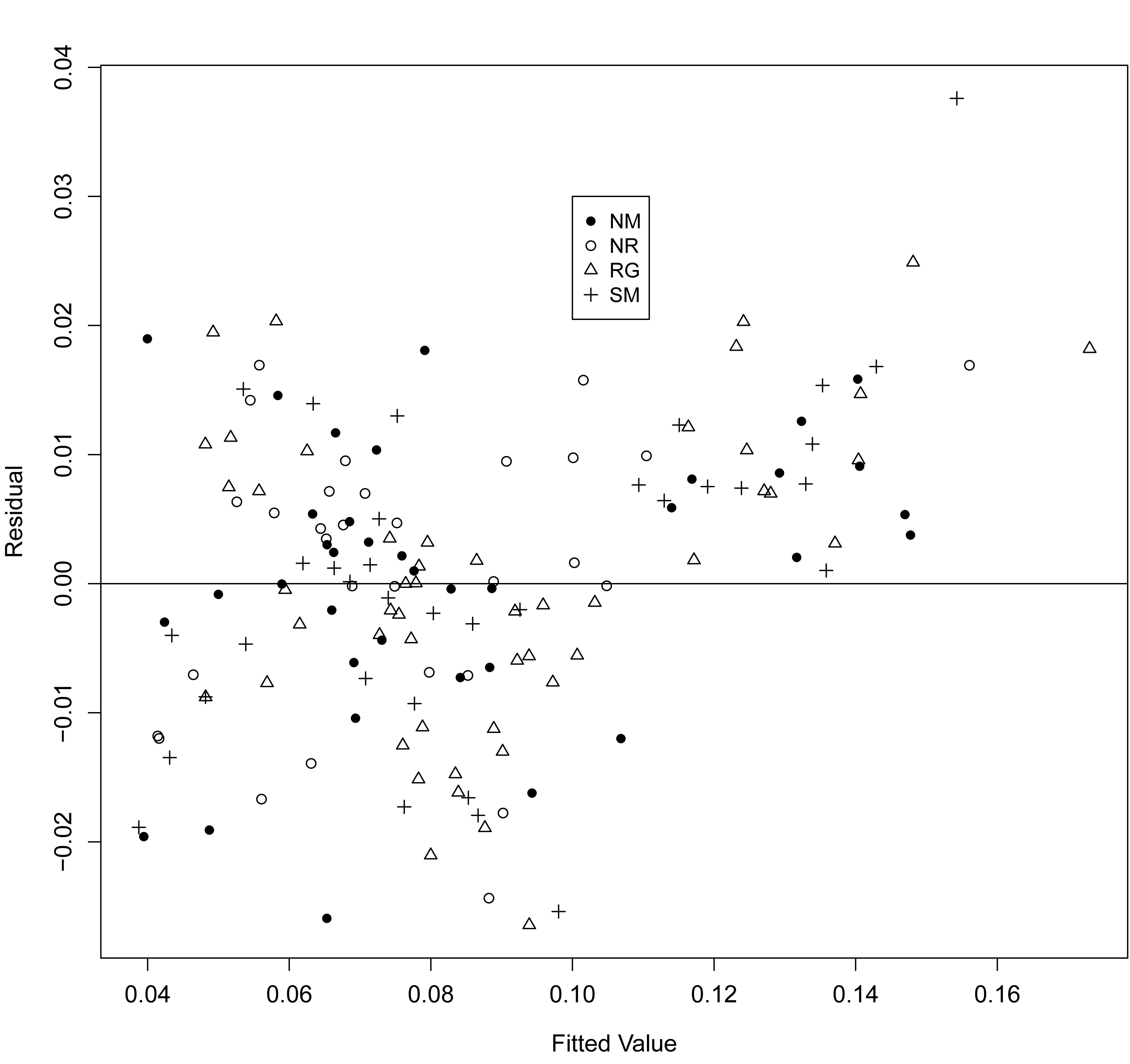


Fig. 3. Fitted values versus residuals from hierarchical beta regression fit to percent body fat of mule deer in the Piceance basin of Colorado. Residuals are from best model as determined by DIC (deviance information criteria).
